# Feasibility of implementing a supervised telehealth exercise intervention in frail survivors of hematopoietic cell transplantation: a pilot randomized trial

**DOI:** 10.1186/s12885-023-10884-5

**Published:** 2023-05-01

**Authors:** Kyuwan Lee, Justin Shamunee, Lanie Lindenfeld, Elizabeth Ross, Lindsey Hageman, Mina S. Sedrak, F. Lennie Wong, Ryotaro Nakamura, Stephen J. Forman, Smita Bhatia, Saro H. Armenian

**Affiliations:** 1 Division of Outcomes Research, Department of Population Sciences, City of Hope Comprehensive Cancer Center, Duarte, CA 91010 USA; 2 Institute for Cancer Outcomes and Survivorship, University of Alabama at Birmingham, Birmingham, AL 35233 United States; 3 Department of Medical Oncology and Therapeutics Research, City of Hope Comprehensive Cancer Center, Duarte, CA 91010 USA; 4 Department of Hematology and Hematopoietic Cell Transplantation, City of Hope Comprehensive Cancer Center, Duarte, CA 91010 USA

**Keywords:** Telehealth exercise, Physical function, Frailty, Hematopoietic cell transplantation

## Abstract

**Background:**

Patients undergoing hematopoietic cell transplantation (HCT) are at high risk of chronic health complications, including frailty and physical dysfunction. Conventional exercise programs have been shown to improve frailty in other cancer populations, but these have largely been based out of rehabilitation facilities that may act as geographic and logistical barriers. There is a paucity of information on the feasibility of implementing telehealth exercise interventions in long-term HCT survivors.

**Methods:**

We conducted a pilot randomized trial to assess the feasibility of an 8-week telehealth exercise intervention in 20 pre-frail or frail HCT survivors. Participants were randomized to either a telehealth exercise (N = 10) or delayed control (N = 10). We administered a remote physical function assessment at baseline, followed by an 8-week telehealth exercise intervention (30-60 min/session, 3 sessions/week), and post-intervention. The primary endpoint was feasibility as determined by 1) > 70% of participants completing all remote physical functional assessments, and 2) > 70% of participants in the exercise group completing > 70% (17/24) of the prescribed exercise sessions. Exploratory outcomes included changes in gait speed, handgrip strength, and short physical performance battery.

**Results:**

The mean [standard deviation] age at study enrollment was 64.7 [9.1] years old. Twelve had undergone allogenic and 8 had undergone autologous HCT at an average of 17 years from study enrollment. Both feasibility criteria were achieved. Nineteen patients (95%) completed all remote study outcome assessments at baseline and post-intervention, and nine participants in the exercise group completed > 70% of prescribed exercise sessions. Overall, no significant group x time interaction was observed on handgrip strength, fatigue, body mass index, and short physical performance battery test (P < 0.05). However, there were significant within-group improvements in four-meter gait speed (+ 13.9%; P = 0.004) and 5-minute gait speed (+ 25.4%; P = 0.04) in the exercise group whereas non-significant changes in four-meter gait speed (-3.8%) and 5-minute gait speed (-5.8%) were observed after 8 weeks.

**Conclusion:**

Implementing an 8-week telehealth exercise intervention for long-term HCT survivors was feasible. Our findings set the stage for innovative delivery of supervised exercise intervention that reduces the burden of frailty in HCT survivors as well as other at-risk cancer survivors.

**Trial registration:**

The protocol and informed consent were approved by the institutional IRB (IRB#20731) and registered (ClinicalTrials.gov NCT04968119; date of registration: 20/07/2021).

## Background

Advances in hematopoietic cell transplantation (HCT) have led to marked improvements in the survival of patients with hematological malignancies [1]. Despite these improvements, long-term HCT survivors remain at high risk for chronic health complications, including physical disability and frailty [2]. HCT survivors are 8.4 times more likely to be frail when compared with age- and sex-matched siblings, and frailty is associated with a nearly three-fold higher risk of premature mortality when compared to non-frail HCT survivors [3].

Exercise is an established strategy to decrease the risk of frailty in conventionally-treated cancer patients [4–6]. To date, exercise-based interventions have largely been medical center-based, which may not be feasible or sustainable for long-term survivors [7–9]. This is especially relevant for survivors who may have geographic constraints or those who are frail and may not be independent enough to travel to the medical center [10–12]. Thus, novel approaches are needed to overcome the limitations of conventional exercise interventions. Recent advances in technology have greatly facilitated the delivery of remote exercise interventions in cancer patients and survivors [13–16]. However, these approaches have largely focused on increasing physical activity participation and have not included objective assessments of physical performance, as would be done during traditional in-person exercise interventions. Moreover, previous approaches have not integrated personalization of exercise delivery and real-time coaching, as would be done during in-person exercise training.

In the general population, there is increased recognition about the importance of telehealth exercise interventions that can provide real-time supervision, goal setting, and performance feedback during exercise sessions [17]. These goals can be accomplished by incorporating automated physiologic data collection assessed at the patient’s home, using existing communication technology (e.g. smartphone or tablets) and biosensors [18, 19]. This new approach to exercise-based intervention has the potential to initiate a paradigm shift toward incorporating telehealth into cancer rehabilitation care delivery. Studies in patients with heart failure have shown that a telehealth exercise intervention is equivalent to center-based cardiac rehabilitation, improving physical function and managing risk factors such as blood pressure and lipid profile [20]. However, despite strong evidence supporting the safety and effectiveness of telehealth exercise interventions, there is a paucity of information on the feasibility of implementing a telehealth exercise intervention in prefrail or frail HCT survivors.

The purpose of this trial was to evaluate the feasibility of a supervised telehealth exercise intervention aimed at improving physical function in prefrail or frail HCT survivors. We hypothesized that an 8-week telehealth exercise intervention would be feasible and improve physical function in the telehealth exercise group.

## Methods and analysis

Study participants were identified from the Blood or Marrow Transplant Survivor Study (BMTSS), which is a retrospective cohort study of patients who received HCT at City of Hope (COH), University of Minnesota, or University of Alabama at Birmingham (UAB) for hematologic malignant diseases, or severe aplastic anemia, and survived at least 2 years after HCT [3, 21, 22]. Eligibility requirements for the current trial included: 1) ≥ 18 years of age at the time of study enrollment; 2) ≥ 2 years from HCT and in clinical remission; 3) self-reported as pre-frail or frail on the BMTSS questionnaire using the following criteria: clinically underweight (body mass index < 18.5 kg/m^2^); exhaustion (self-report of feeling tired); low energy expenditure (self-report of physical activity for < 2 days/week); slowness (self-reported limitations in climbing stairs or walking 1 block); and weakness (self-report of weakness in movement), with the presence of 2 of the indices classified as prefrail and ≥ 3 indices classified as frail [3]; 4) able to provide written informed consent; 5) physically able and willing to complete all study procedures; 6) English-speaking. Exclusion criteria included: (1) clinically significant/active cardiovascular disease (e.g., unstable angina, uncontrolled arrhythmia, cardiomyopathy); (2) contraindications to exercise (e.g. acute infectious disease); (3) recovering from a recent injury or were physically injured in the 6 months prior to approach for enrollment; (4) already participating in ongoing structured exercise (> 60 min/week); (5) females who were pregnant or planning to become pregnant.

### Study procedures

**Fig. 1:**
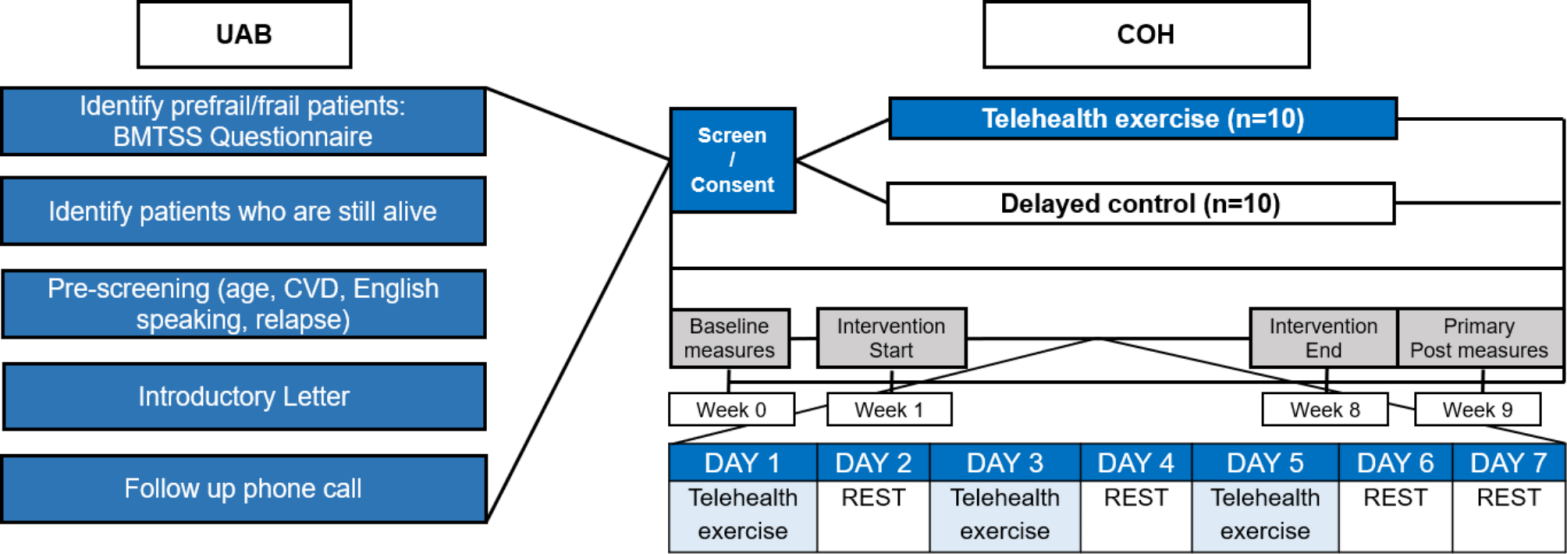
Study schema Abbreviations: BMTSS: Blood or Marrow Transplant Survivor Study; COH: City of Hope; CVD: Cardiovascular disease, UAB: University of Alabama Birmingham

Information on frailty status was obtained from questionnaires completed by BMTSS participants [3]. Following a database review for eligibility, research staff at UAB sent an introductory mail to ask if participants would agree to be contacted by COH (**Study Schema**, Fig. 1). If BMTSS participants were interested in the study, they were referred to research staff at COH who confirmed inclusion/exclusion criteria by phone, and consented eligible survivors to study participation via a web-based platform (DocuSign™; San Francisco, CA). Participants were then randomly assigned to the telehealth exercise group (n = 10) or delayed control group (n = 10). Our study biostatistician conducted randomization using computer-generated, investigator-blinded randomization (Parallel 1:1). Survivors with active chronic graft versus host disease (GvHD) were required to have medical clearance by their physician to participate in the study.

### Remote assessments of frailty and physical function

The primary endpoint was feasibility as determined by 1) > 70% of participants completing all remote physical functional assessments, and 2) > 70% of participants in the exercise group completing > 70% (17/24) of the prescribed telehealth exercise sessions. Study participants were mailed a set of 3 gait sensors (for hip, right foot, and left foot), a hand dynamometer, measuring tape, and five exercise elastic bands that varied by resistance level. They completed an initial technology instructional support session via video conferencing. We used the telehealth exercise platform developed by Moerum Technologies (moterum.com, Salt Lake City, UT), which enables implementation of remote exercise strategies accessible through digital platforms (e.g. real-time video conferencing on smart phones or tablets) and customizable to individual needs.

The secondary endpoint was frailty characteristics as assessed using a 5-scale frailty index before and after the 8-week period: body mass index (BMI), fatigue (13-item Functional Assessment of Chronic Illness Therapy [FACIT] fatigue scale), [23] energy expenditure, [3] gait speed, [24] and handgrip strength [25]. BMI, fatigue, and energy expenditure were self-reported. In addition, Short Physical Performance Battery (SPPB) was assessed at baseline and post-intervention, which included three lower extremity standing positions completed on the ground for 10 s or when the participant steps out of position with (a) both feet side-by-side stand, (b) semi-tandem stand with the side of the heel of one foot touching the big toe of the other foot, and (c) tandem stand with the heel of one foot touching the big toe of the other foot placed [26]. We assessed gait speed by instructing participants to measure and mark a 4-meter flat surface distance, across which they were asked to walk at their usual pace while time was recorded using an electronic timer. In addition to the 4-meter gait speed, we also assessed habitual gait speed using gait sensors, [24] instructing participants to wear gait sensors and walk as usual for 5 min. We assessed chair stand under two conditions: (a) perform a single chair stand; (b) perform five repeated chair stands as quickly as possible; time to completion was recorded. Handgrip strength was measured twice using a hand-held dynamometer and the amount of maximal force exerted on the dynamometer for both non-dominant and dominant hand were documented and averaged.

### Exercise intervention

The 8-week telehealth exercise intervention (> 30 min per session; 3 sessions/week for 8 weeks) began within 3 weeks of baseline study assessments. Exercise programs were individualized and prescribed based on participants’ baseline assessment, physical limitations, and exercise preferences. Exercise intensity progression was achieved by altering the color of resistance bands. If a participant reached a band color with the max resistance, an additional band was added, allowing them to use up to all 5 bands at once. Each exercise session consisted of exercises targeting four essential components (dynamic balance, strength, core stability and postural control) [27]. If a participant was unable to complete the exercises as planned, the exercise trainer provided alternative options and exercise modifications. Participants were also offered the option to reschedule or make up an exercise session if they were unable to attend a session on the planned date. Exercise adherence for each participant was captured on the Moterum platform and extracted to assess the feasibility of the prescribed exercise program. The same exercise program was offered to the participants in the delayed control group after the 8-week follow-up period.

### Sample size and statistical analysis

The sample size was evaluated using the statistical recommendation for the standardized effect size of 0.8 in 10 participants each arm [28]. Information obtained from the current study may provide prevalence estimates to guide a larger study. We had originally planned to enroll 24 participants accounting for 20% voluntary attrition rate over time. However, recruitment was terminated early because all study participants were retained for the eight-week study duration, except for one participant in the delayed control group who died due to causes that were unrelated to the study. We generated descriptive statistics for participants’ demographics, treatment history, and outcome measures. The study was considered feasible if 1) > 70% of participants successfully completed all remote physical functional assessments, and 2) > 70% of participants in the exercise group completed > 70% (17/24) of the prescribed exercise sessions. Exploratory outcomes of interest included physical function and frailty measures, as assessed by SPPB, 5-minute gait speed (gait sensors), 13-item FACIT-fatigue scale, handgrip strength, and self-reported energy expenditure. Participants were considered to have a clinically meaningful improvement if they demonstrated increases in: ≥1 point for SPPB, or ≥ 0.1 m/s increase for gait speed, or ≥ 1 kg increase in handgrip strength [29]. For within group difference, the changes in physical function from baseline to week 9 were examined by a paired t-test, with a level of significance set at P < 0.05. Repeated measures ANOVA on the trial outcomes was a 2 (group: telehealth exercise, delayed) x 2 (time: baseline, post-intervention) analysis.

## Results

**Fig. 2:**
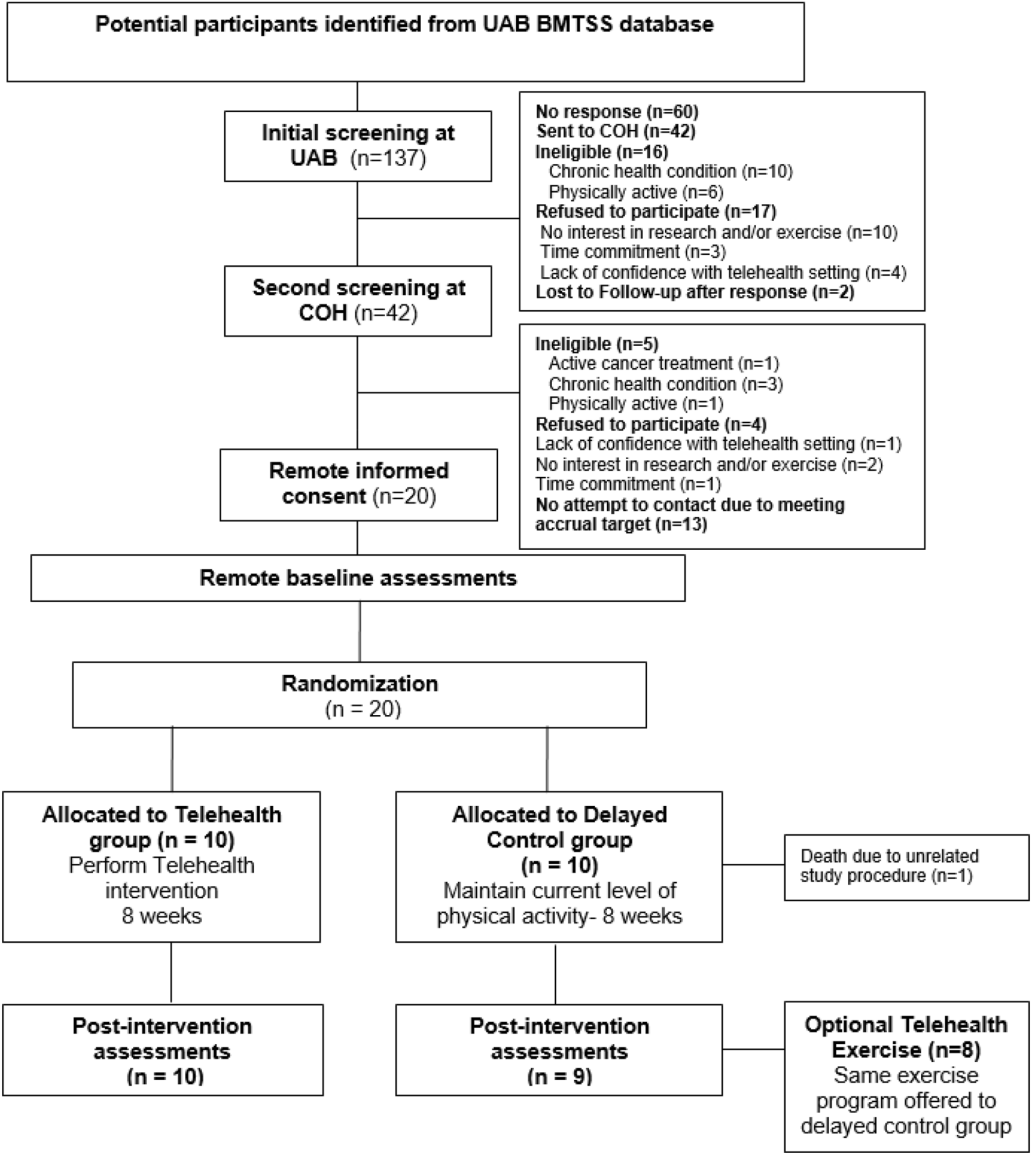
CONSORT diagram of telehealth exercise intervention Abbreviations: BMTSS: Blood or Marrow Transplant Survivor Study; COH: City of Hope; UAB: University of Alabama Birmingham

There were 137 self-reported prefrail/frail HCT survivors identified in the BMTSS cohort. Of the 75 self-reported prefrail/frail survivors who were successfully contacted, 17 refused the referral, and 16 were deemed ineligible; Fig. 2. The entire study period was from July 20th, 2021 to August 15th, 2022. Overall, 42 (71% of contacted and eligible) survivors were referred to COH and 20 were eventually consented to the trial; Fig. 2. Among the 20 study participants, 19 were retained over the 8-week intervention and one participant in the delayed control group died due to causes that were unrelated to the study. One participant in the exercise group developed a lung infection but retained during the 8-week intervention period, resulting in < 70% participation to the prescribed exercise regimen (15/24 sessions). This trial was ended when the target accrual (n = 20) was achieved, and the follow-up assessment was completed.

Baseline characteristics are presented in Table 1. The mean [standard deviation] age at study enrollment was 64.7 [9.1] years old; 50% were female, 73% were non-Hispanic White. Study participants had undergone HCT for Hodgkin or Non-Hodgkin lymphoma (45%), acute/chronic leukemia (40%), myelodysplastic syndrome (10%) and multiple myeloma (5%). Twelve (60%) had undergone allogenic HCT and 8 (40%) had undergone autologous HCT. Mean time from HCT was 17 years. Eighteen participants (90%) were classified as frail and 2 were classified as prefrail. Among 12 patients treated with allogenic HCT, 9 patients had chronic GvHD. Low energy expenditure (100%) and high level of fatigue (95%) were the most common frailty characteristics, followed by slow gait speed (80%), weakness (75%), and low BMI (20%).

**Table 1:** Baseline Participant Characteristics

	All (N = 20)	Telehealth Exercise group (N = 10)	Delayed control Group (N = 10)
**Age at study enrollment, years, mean (SD)**	64.7 (9.1)	64.8 (10.6)	64.7 (7.9)
**Sex** Male Female	10 (50) 10 (50)	4 (40) 6 (60)	6 (60) 4 (40)
**Transplant type** Allogeneic Autologous	12 (60) 8 (40)	6 (60) 4 (40)	6 (60) 4 (40)
**Race/ethnicity** Non-Hispanic white Hispanic white African American Asian/Pacific Islander	13 (65) 2 (10) 2 (7) 0 (0)	7 (70) 1 (10) 0 (0) 1 (10)	6 (60) 1 (10) 2 (14) 1 (10)
**Cancer Diagnosis** ALL AML CLL CML MM MDS HL NHL	1 (5) 3 (15) 1 (5) 3 (15) 1 (5) 2 (10) 2 (10) 7 (35)	1 (10) 2 (20) 0 (0) 2 (20) 0 (0) 1 (10) 1 (10) 3 (30)	0 (0) 1 (10) 1 (10) 1 (10) 1 (10) 1 (10) 1 (10) 4 (40)
**Chronic GvHD** Yes No	9 (45) 11 (55)	5 (50) 5 (50)	4 (40) 6 (60)
**Frailty status** Frail Pre-frail	18 (90) 2 (10)	9 (90) 1 (10)	9 (90) 1 (10)

Note. Data are presented as No. (%) in each column unless otherwise indicated. Abbreviations: ALL, Acute lymphocytic leukemia; AML, Acute myeloid leukemia; CLL, Chronic lymphocytic leukemia; CML, Chronic myeloid leukemia; MM, Multiple myeloma, MDS, Myelodysplastic syndromes; HL, Hodgkin’s lymphoma; NHL, Non-Hodgkin’s lymphoma, SD, standard deviation

### Study assessments and adherence to prescribed exercise sessions

Nineteen patients (95%) successfully completed all remote physical function assessments at pre- and post-intervention, and nine participants (90%) in the exercise group completed > 70% prescribed exercise sessions. The mean adherence to the 24 prescribed sessions for 10 patients in the exercise group was 94.2% (226/240 sessions); 9 of 10 participants attended at least 23 of 24 sessions. Although we remained flexible with scheduling and adapted to participants’ availability including weekends, most participants chose to perform exercise on weekdays (e.g. Monday/Wednesday/Friday or Tuesday/Thursday/Friday), except 2 participants who chose to include one weekend day for each week. No serious adverse events or unintended effects were associated with the exercise intervention during the 8 weeks.

### Physical function outcomes

Table 2 includes baseline and post-intervention (8 weeks) physical function outcomes. Overall, there was no group x time interaction on physical function outcomes of interest. Within-group comparisons are shown below.

**Table 2:** Frailty Characteristics

Outcomes	Baseline, mean (SD)	Post-intervention, mean (SD)	
	Mean (SD)	Mean (SD)	*P*
**Short Physical Performance Battery** Telehealth Exercise Delayed Control	10.0 (2.0) 8.2 (2.4)	10.4 (2.2) 7.8 (3.8)	0.63 0.69
**4-meter Gait Speed (m/s)** Telehealth Exercise Delayed Control	0.86 (0.22) 0.78 (0.27)	0.98 (0.25) 0.75 (0.23)	**0.004** ^*^ 0.11
**5-minute Gait Speed (m/s)** Telehealth Exercise Delayed Control	0.63 (0.30) 0.68 (0.24)	0.79 (0.24) 0.64 (0.27)	**0.04** ^*^ 0.13
**Handgrip Strength (Dominant arm)** Telehealth Exercise Delayed Control	28.7 (11.7) 26.6 (6.7)	30.3 (14.0) 23.9 (10.6)	0.15 0.51
**Handgrip Strength (Non-Dominant)** Telehealth Exercise Delayed Control	28.9 (12.2) 23.9 (10.7)	29.6 (11.6) 22.5 (10.2)	0.48 0.70
**Fatigue** Telehealth Exercise Delayed Control	39.2 (6.6) 31.7 (11.3)	40.7 (5.6) 31.3 (9.9)	0.19 0.69
**Body mass index** Telehealth Exercise Delayed Control	29.3 (6.9) 27.1 (6.7)	29.7 (9.7) 26.5 (6.4)	0.89 0.08

#### Gait speed

Four-meter gait speed was significantly improved (0.86 ± 0.22 to 0.98 ± 0.25 m/s; P = 0.004) in the exercise group, while there was no significant change in the delayed control group (0.78 ± 0.27 to 0.75 ± 0.23 m/s, P = 0.11). However, there was no group x time interaction between the two groups before and after 8 weeks (P > 0.05). Notably, 8 out of 10 patients in the exercise group increased gait speed > 0.1 m/s, whereas only one participant in the delayed control group increased gait speed by > 0.1 m/s. Five-minute gait speed, as measured by gait sensors, also increased significantly in the exercise group (0.63 ± 0.30 to 0.79 ± 0.24 m/s: P = 0.04), and there was no significant change in the control group (0.68 ± 0.24 to 0.64 ± 0.27 m/s: P = 0.13).

#### Handgrip strength

There was no group x time interaction between the two groups before and after 8 weeks. Overall, there was a slight improvement in dominant arm handgrip strength in the exercise group, but it did not reach statistical significance (28.7 ± 11.7 to 30.3 ± 14.0 kg; P = 0.15). However, 5 out of 10 participants increased > 1 kg of handgrip strength (dominant arm) following the 8-week exercise training, compared to one participant in the control group. There was no significant change in the control group (P > 0.05).

#### SPPB

At baseline, mean SPPB score was 10.0 ± 2.0 for the exercise group and 8.2 ± 2.4 for the delayed control group. There was no significant mean difference between the two groups before and after 8 weeks. Of note, among 4 participants who had an SPPB score < 10 at baseline in the exercise group, 3 participants increased SPPB (≥ 1 point) by the end of the 8-week intervention. The remaining 6 participants who had SPPB ≥ 10 maintained the same SPPB. In the delayed control group, 2 participants increased SPPB by 1 point, 5 participants maintained the same SPPB, and 2 participants reduced their SPPB by 1 point after 8 weeks.

#### Fatigue

There was no significant mean difference between the two groups before and after 8 weeks in the exercise (39.3 ± 6.6 to 40.7 ± 5.6) and control (31.7 ± 11.3 to 31.3 ± 9.9) groups; P > 0.05.

## Discussion

This study is the first to demonstrate the feasibility of implementing a fully remote outcome assessment and delivery of personalized real-time exercise coaching in a high-risk population. We found that an 8-week telehealth exercise intervention (3 sessions/week) is feasible and safe, based on the high compliance to the intervention without any serious adverse events. This finding is important because specialized cancer centers are not widely distributed geographically, and access to exercise rehabilitation facilities may be limited by proximity [30]. Of note, 95% of our study participants were frail, which may have further limited mobility and survivors’ ability to travel to exercise facilities regularly (i.e. 3 times a week). As patients and clinicians report high satisfaction with the use of telehealth, [31, 32] rehabilitation facilities may utilize telehealth approaches to provide exercise rehabilitation programs with those patients experiencing physical and/or geographic restrictions.

Overall, there was no significant group x time interaction on physical function outcomes and patient-reported outcomes. However, we believe our preliminary evidence of within group improvement in gait speed and handgrip strength in the exercise arm is clinically important given the association between reduced gait speed/higher survival (27% increased mortality per 0.1 m/s decrease), [33] and increased handgrip strength/reduced overall mortality (4% reduced mortality per 1 kg increase) [34]. Based on its simplicity and predictive value, the assessment of gait speed and grip strength has been widely used to characterize the severity of frailty, and have been used to demonstrate the efficacy of in-person exercise interventions in HCT patients and survivors at varying timepoints after HCT [35–38]. For example, Knols et al. (2011) showed that a 12-week supervised in-person exercise intervention (2 session/week) significantly improved gait speed (9.5% increase) in 64 patients within the first 6 months of HCT [36]. Another study in 33 patients also showed that resistance exercise training could maintain gait speed (17% worsening in the control group) and strength (8% worsening in the control group) after HCT (duration after HCT not specified). [35]. Collectively, these data and ours suggest that resistance exercise training is an important rehabilitation strategy to improve gait speed and strength during HCT survivorship, and highlight the opportunity for telehealth exercise to successfully address the limitations of in-person exercise strategies with comparable efficacy. Our study extends the experience from previous studies in HCT by demonstrating the feasibility of fully remote collection of gait speed and handgrip strength in HCT survivors, and by focusing on a very long-term (mean 17 years from HCT) survivor population that has historically been underrepresented in exercise-based intervention studies.

In the current study, we did not observe a statistically significant change in SPPB or 13-item FACIT-fatigue scale. With regard to SPPB, it is worth noting that 60% of participants in the exercise group had SPPB 10 or above, which is generally considered a normal score [39]. In our study, SPPB increased in individuals with a score < 10, but efficacy was limited in individuals with SPPB ≥ 10, possibly due to the ceiling effects of exercise for individuals without SPPB-defined functional impairment [40, 41]. In contrast, the effects of exercise on fatigue are not well studied in HCT survivors. Particularly, the cutoffs for 13-item FACIT fatigue scale are not clearly understood in this population, making the interpretation of data more challenging [39]. Nevertheless, beneficial effects of exercise on fatigue were generally demonstrated in other cancer populations including breast, [42] lung, [43] colorectal cancers, [44] that utilized aerobic exercise with longer interventions (e.g. 6 months), representing an important direction for future research in HCT survivors.

The strengths of this study included: (1) specifically targeting prefrail/frail HCT survivors, a group at high risk for subsequent mortality, (2) no in-person visits, which reduced travel-related time/cost of participants, (3) no need for specialized exercise equipment, other than resistance bands, contributing to the low cost of exercise implementation, (4) high adherence rate due to adoption of flexible telehealth exercise schedules, (5) ability to deliver individualized exercise prescriptions, including exercise type/intensity/time based on participant’s physical status and perceived ratings of exertion.

Despite the strengths, our study has several limitations. First, the small sample size limits our ability to comment on the true efficacy of the intervention for the broader HCT survivorship population. Larger studies are needed to address this limitation, and the current study paves the way for their development. Second, we did not perform comprehensive phenotyping to determine changes in body composition, which may have provided additional insights into changes in muscle mass and frailty over time. Third, we acknowledge that technology-based interventions such as ours require the availability of exercise trainers, biosensors, and mobile technologies to deliver the intervention, which may reduce the generalizability of the trial findings. However, these initial costs may be offset by reducing the costs associated with in-person clinic visit, travel to exercise facilities (e.g. transportation, parking) and specialized center-based exercise equipment [45]. Additional studies are needed to evaluate the cost-effectiveness of using biosensors and telehealth exercises in this population.

In conclusion, we demonstrate that a remotely delivered supervised telehealth exercise strategy is safe, and may be efficacious. This study provides preliminary and much-needed evidence to facilitate the development of comprehensive telehealth exercise programs in HCT survivors. Future studies will need to integrate participant feedback to help tailor exercise-based interventions to a more diverse group of survivors, and to evaluate the impact of social determinants of health (e.g. education, income, marital status, built environment) on participation as well as retention. The growing population of HCT survivors (estimated > 500,000 by 2030) [46] emphasizes the need to continue to invest in larger scale telehealth exercise intervention studies to establish the efficacy, long-term sustainability, and cost effectiveness of remotely delivered interventions across geographically and demographically diverse HCT populations.

## Data Availability

The datasets used and/or analyzed during the current study are available from the corresponding author on reasonable request.
